# Health and Functioning in Grandparents After a Young Grandchild’s Death

**DOI:** 10.1007/s10900-015-0018-0

**Published:** 2015-03-29

**Authors:** JoAnne M. Youngblut, Dorothy Brooten, Kathleen Blais, Colleen Kilgore, Changwon Yoo

**Affiliations:** 1 Nicole Wertheim College of Nursing and Health Sciences, Florida International University, AHC 3, Rm 241, 11200 SW 8th St., Miami, FL 33199 USA; 2 South University, West Palm Beach, FL USA; 3 Stempel College of Public Health and Social Work, Florida International University, Miami, FL USA

**Keywords:** Grandchild death, Grandparents, Grief, Depression, PTSD

## Abstract

This cross-sectional study examined the physical and mental health, grief and role functioning of 136 grandparents in the first year after death of their young grandchild (newborn through 6 years). Grandparents were 36–77 years old; 73 % female; 24 % Hispanic, 38 % Black/African American, and 38 % White. Mean age of the 115 deceased grandchildren was 12.8 months (*SD* = 20.71) with 37 % <1 month old; 65 % were male, 77 % died in the hospital. Grandparents were recruited through state death records and interviewed by telephone. Grandparents experienced: clinical depression (31 %), PTSD (35 %); illnesses (28 %), hospitalizations, new chronic health conditions (mental disorders, hypertension, angina, cancer), and medication changes. Grandparents who provided care for the deceased grandchild had more intense symptoms of grief, depression and PTSD and more trouble focusing at their jobs. Severity of depressive and/or PTSD symptoms were more likely to be at clinically important levels for grandparents who had provided childcare for the deceased grandchild than for non-caregiving grandparents. Black grandparents had more severe symptoms of PTSD and thought more about their deceased grandchild on the job than White grandparents. The interaction effect of race/ethnicity and provision of child care was significant for PTSD and Blame and Anger. Hispanic grandparents who provided some child care for their deceased grandchild had less severe PTSD symptoms than caregiving Black and White grandparents. Caregiving Hispanic grandparents also experienced less Blame and Anger than White caregiving grandparents.

## Introduction

Death of a grandchild is devastating for most grandparents. It is loss of a grandparent’s joy with their grandchild at major life events, loss of what this grandchild might have become and loss of a portion of their own legacy. But it is more. Grandparents experience great pain for their adult child (grandchild’s parent) whom they try to support while grieving themselves for the deceased grandchild. They also support their adult child’s and the deceased grandchild’s siblings and yet have difficulty finding a place of support and solace for themselves. Li et al. [1–4] reported increases in stress-related health outcomes including cancer, heart disease, mental disorders and deaths for parents 1–5 years after their child’s death. These health-related effects have not been studied in grandparents following a grandchild’s death, despite the multiple stressors they experience surrounding the death. It is unclear whether health outcomes differ for grandparents who provided care for this grandchild, a growing grandparent responsibility. Differences in grandparents’ health and functioning by race/ethnicity after a grandchild’s death are unknown despite the growth in minority populations, especially Hispanics [5]. Despite these changing factors, research on grandparent health and functioning after a grandchild’s death over the last 25 years is very sparse.

The purpose of this cross-sectional study was to examine: grandparents’ physical and mental health, grief, and role functioning (employee, spouse/partner) in the first year after a young (newborn through 6 years) grandchild’s death and whether these outcomes differ by race/ethnicity and level of care provided to the deceased grandchild.

### Young Child Deaths and Grandparent Research

More than 34,000 children (newborn through 14 years) died in the US in 2010; 72 % were infants <1 year old, 13 % were 1–4 years old, and 15 % were 5–14 years old [6]. More deceased infants/children under 5 were White (n = 13,145) than Black (n = 8044) or Hispanic (n = 6097). However, the death rate per 100,000 for Black infants (1167.2) was more than double that for White (528.2) and Hispanic infants (510.4).

The nine studies published since 1991 on grandparents’ responses to grandchild death—grief, depressive symptoms, and sometimes spouse/partner or adult child relationships—are mostly qualitative. Few quantify effects on grandparent mental health, and none focus on physical health and role functioning. Of these nine studies, five were conducted in the US and one each in Africa [7], Canada [8], Ireland [9], and Israel [10]. Four of the five US studies had samples of primarily White grandparents [11–14]; the fifth did not report race/ethnicity [15]. Research on racial/ethnic differences for grandparents whose young grandchild dies is lacking, despite research findings of differences in the health of bereaved parents by race/ethnicity [16].

Most research on the aftermath of a child’s death focuses on parents [14, 17, 18]. Studies find negative effects on bereaved parents’ physical health (newly diagnosed cancer, diabetes, hypertension, angina [16], greater risk of death [4]) and mental health—depression, suicidality, and post-traumatic stress disorder (PTSD) [16, 19]. Research on bereaved parents’ role functioning as parents, partners, and employees is limited and findings are conflicting.

### Grandparent Responses

Grandparents describe a grandchild’s death as the most difficult situation they have ever experienced [20]. Although loss of relatives and friends is inevitable as one ages, grandparents do not expect to endure a grandchild’s death. Bereaved grandparents often feel forgotten by others who are important to them [10] and have been overlooked in research.

Grandparents grieve physically and psychologically after their grandchild’s death [7, 9]. Their primarily negative responses include disbelief that the grandchild died before them, helplessness, exhaustion, bitterness, somatic symptoms, pain, anxiety, depression, PTSD, increased alcohol and drug use, and thoughts of suicide [13, 21]. Roose and Blanford [14] found that grandparents reported pain for their grieving adult child (grandchild’s parent) plus the pain of losing their grandchild. In one qualitative study [9], grandparents reported pain during the grandchild’s illness and post-death regarding their lost hopes and dreams for this grandchild’s future. They found grandparents experienced pain not only from loss of their grandchild and their adult child’s grief, but also from other sources, resulting in what these investigators [9] call “cumulative pain”. Yet, despite their pain, bereaved grandparents provided the most support to their adult child [20].

Grandparents reported symptoms suggesting the grandchild’s death had an impact on their physical and/or mental health. In a sample of 80 grandparents interviewed up to 12 years after their grandchild’s death [15], about half reported flashbacks of the death and felt their memories and grief would not fade over time. Some grandparents (42 %) wanted to sleep until the pain was over. Others considered suicide and reported increased alcohol and drug use. One grandparent hospitalized with pneumonia soon after the funeral attributed his condition to being upset by the grandchild’s death. Grandparents in another study [13] experienced physical symptoms when told about the grandchild’s death. More grandmothers reported numbness, shock, disbelief, and physical symptoms than grandfathers. Grandparents in Fry’s study [8] reported their world was shattered. Some wished they could take the grandchild’s place in the grave. One grandmother reported that her spouse seemed to have stopped living his life and now simply existed [10].

From support group sessions with 10 bereaved Israeli grandparents (ages 45–75), Nehari et al. [10] found that grandparents’ feelings and symptoms were similar to those expressed by bereaved parents. Grandparents needed to talk about their deceased grandchildren but had difficulty finding someone to listen. Grandparents often felt isolated and emotionally distant from their adult child (grandchild’s parent) and found it challenging to deal with the grandchild’s parents and siblings who were at different places in their grief. Nehari et al. [10] note that, although there is a culture of bereavement for parents and siblings, no such culture exists for grandparents. When excluded from family bereavement activities, grandparents voiced concern about the legitimacy of their grief and not finding a “space” for their grief.

### Grandparents Providing Childcare

Many US grandparents live with and/or provide care for at least one grandchild, which may affect grandparent response to grandchild death. According to the US Census Bureau [22], almost 7 million grandparents live with at least one grandchild younger than 18. Of the almost 3 million grandparents who live with and are responsible for their grandchildren, 2 million are 30–59 years old and .9 million are 60 years of age and older. More White grandparents (63 %) lived with their grandchildren than Black (19 %) or Hispanic (25 %) grandparents. However, a greater proportion of Black live-in grandparents (49 %) were responsible for their grandchildren than White (41 %) or Hispanic (32 %) grandparents. Some grandparents do not live with their grandchildren but take care of them either regularly or episodically [22]. Studies on grandparents raising grandchildren almost exclusively have included healthy grandchildren. Research on caregiving grandparents when the grandchild has an acute, chronic, or life-threatening illness is minimal and rarely considers the grandparents’ race/ethnicity or their level of caregiving.

The influence of having provided custodial or non-custodial care for the deceased grandchild on grandparents’ grief, health, and role functioning has not been studied. Grandparents may provide non-custodial care for their grandchildren to help the parent (their adult child) with divorce, single parenthood, and the cost of child care [23]. Custodial care often falls to grandparents when the parent(s) have died or been incarcerated, have abandoned their children, or are no longer able to take care of them [24, 25].

### Grandparent Role Function

Studies of grandparents’ employment after a grandchild’s death have not been reported. The Society for Human Resource Management [26] found that all of the 507 human resource professionals in its study reported that their employer provided leave for the death of a child, parent, or spouse/partner. However, none reported their employers having a policy of paid leave for grandchild death. In Youngblut et al.’s study [16] of 249 bereaved parents, half of the mothers returned to their jobs within 32 days of their infant’s/child’s death. Half of the fathers returned to their jobs within 14 days. However, three mothers and four fathers took no time off after the death. Timing of grandparents’ return to their jobs is not known.

For some grandparents, the grandchild’s death had an impact on their relationship with their spouse/partner. In their study of 74 parents and grandparents whose child or grandchild died, Arnold et al. [27] found that effects on marital relationships varied with the status of participants’ grief. Those who said their grief had ended (36.5 %) reported their marital relationship was the same or better since the death. The 63.5 % who continued to grieve reported their sex lives and health had worsened and their relationship and communication with their spouse/partner was strained. DeFrain et al. [15] found that most grandmothers wanted to talk about their grandchild, the death, and their feelings and emotions, but their husbands were silent on these topics. Most grandparents characterized their marriages as the same (68 %) or stronger (29 %); 3 % believed the grandchild’s death had weakened their marriages [15]. In contrast, Arnold et al. [27] in a later study found that almost two-thirds of grieving grandparents reported marital difficulties.

In summary, few studies have focused on grandparents’ health and functioning after a grandchild’s death. In those studies, grandparents experienced negative symptoms, thoughts, and feelings similar to bereaved parents. They reported having trouble believing the grandchild had died, dealing with the grandchild’s parents and siblings, and finding people who “allowed” them to talk about the event. Some wished they had died instead of the grandchild, grieved for their adult child (grandchild’s parent), and felt isolated and distant from their families. However, time since the grandchild’s death (3 months–12 years) and grandchild age at death (newborn to 32 years) varied widely within the primarily White samples. Grandparents were generally recruited through bereavement support groups and flyers or advertisements in stores, senior citizens’ centers, and newsletters of national organizations for people who have lost a child. It is very likely that grandparents recruited from these sources many years after the death of their newborn to adult grandchildren are different than grandparents recruited through official state death records within 12 months of their young grandchild’s death. Differences in grandparent health, functioning, and grief responses after a grandchild’s death by race/ethnicity and grandchild caregiving have not been investigated, despite a growing minority population [5] and large numbers of grandparents providing care for their grandchildren [22].

## Methods

In this cross-sectional study, deceased grandchildren (newborn to 6 years) whose parents resided in south Florida were identified through death records obtained from the Florida Office of Vital Statistics. Hispanic, Black non-Hispanic, and White non-Hispanic grandparents who understood spoken English or Spanish and experienced a grandchild’s death in the previous 1–9 months were eligible to participate. Exclusion criteria were: living in an extended care or skilled nursing facility because of diminished physical and/or cognitive capacity, scored ≤20 on the telephone Mini-Mental Status Examination [28], and/or whose deceased grandchild had been in foster care. Grandparents were also excluded if the grandchild’s parent(s) died in the illness/injury event because they would be dealing with two deaths simultaneously.

### Procedures

The study was approved by the institutional review boards at the university and the Florida Department of Health. The research team complied with APA ethical standards in the treatment of human subjects. Deceased newborns/children through 6 years old (85 % of all deceased US children) [6] were identified through death records. In Florida, names of deceased persons (including children) and the names and contact information for their next of kin, but not cause of death, are public record and available from the Department of Health’s Office of Vital Statistics. The next of kin on the death record was sent a letter (in Spanish and English) explaining the study and encouraging him/her to talk with the grandparents about providing their contact information to the study. One week later, a research assistant (RA) called the next of kin to obtain contact information for eligible grandparents. For families without an available telephone number, the RA visited the next of kin’s address to obtain grandparent contact information. Of the 1712 deceased grandchildren (newborn—6 years) listed in death records, next of kin could not be contacted for 1393 (81 %) grandchildren due to inaccurate addresses, telephone disconnected, wrong number, or no phone. Of the 319 (19 %) next of kin contacted, 144 (45 %) provided grandparent contact information, 126 (40 %) declined, and 49 (15 %) reported the grandparents were ineligible due to: language other than English or Spanish or living outside the US with no phone (n = 37, 76 %), no living grandparents (n = 7, 14 %), or no contact with the grandparents (n = 5, 10 %). A similar contact letter (Spanish and English) then was sent to eligible grandparents. One week later, the RA called the grandparents to further explain the study, obtain their agreement to participate, screen grandparents over 75 with the Telephone MMSE [28], and schedule a time for data collection. Of the 238 grandparents of 144 grandchildren contacted, 159 (67 %) grandparents of 115 (80 %) deceased grandchildren participated. In this study’s subsample, none of the 99 grandmothers and 37 grandfathers was married or partnered with another grandparent in the subsample.

In a 30-min telephone interview, grandparents completed a demographic form including questions on physical health and employment functioning, Hogan Grief Reaction Checklist (HGRC), Beck Depression Inventory (BDI-II), Impact of Events Scale-Revised (IES-R) and Dyadic Adjustment Scale (DAS) (if partnered). Mean time between grandchild death and grandparent interview was 13.7 weeks (SD = 7.68): 31 % at 3–8 weeks, 43 % at 8.1–16 weeks, 21 % at 16.1–26 weeks, and 5 % at 26.1–52 weeks.

## Measures

### Grandparent Health

Grandparents rated their health over the previous 2 weeks compared to others their age on a scale from 1 “poor” to 10 “excellent.” Grandparents reported the number of times they had been ill and/or hospitalized since the grandchild’s death. They indicated presence/absence of a series of chronic conditions, when diagnosed (before or after the grandchild’s death), medications for each condition, and changes in medication post-death.

#### Depression

The BDI-II [29] contains 21 items that grandparents rated on a scale from 0 to 3 for the previous 2 weeks. Possible scores ranged from 0 to 63; higher scores indicate greater severity of depressive symptomatology. Scores used to define the depression severity groups [29] were: 0–13 “none,” 14–19 “mild,” 20–28 “moderate,” and 29–63 “severe.” Coefficient alpha was .92 for grandparents.

#### Post-traumatic Stress Disorder (PTSD)

The IES-R [30] measures PTSD as intrusiveness of thoughts about the grandchild, avoidance of reminders of the grandchild, and hyperarousal. Grandparents rated 22 items from 0 “not at all” to 4 “extremely” to indicate how distressing each had been in the past 2 weeks regarding their grandchild’s death. Possible scores range from 0 to 88. Total scores of 34 and higher indicate presence of PTSD [30]. Coefficient alpha was .94 for grandparents.

### Grandparent Grief

Hogan, Greenfield, and Schmidt [31 p. 17] define the 6 HGRC subscales as: (1) Despair—“feelings of hopelessness, sadness, and missing the loved one;” (2) Panic Behaviors—“physiological characteristics including autonomic nervous system arousal associated with fear;” (3) Blame and Anger—“bitterness, hostility, and vengeful feelings;” (4) Detachment—“avoidance of tenderness, withdrawal from others, and change in identity;” (5) Disorganization—“difficulty concentrating, learning new information, and remembering information;” (6) Personal Growth—“spiritual and existential awareness.” Grandparents rated 61 items from 1 “Does not describe me at all” to 5 “Describes me very well” for the previous 2 weeks. Subscales had 7–14 items. Possible scores are: Despair, 13–65; Panic Behaviors, 14–70; Blame and Anger, 7–35; Detachment, 8–40; and Disorganization, 7–35. Higher scores indicate greater grief. For Personal Growth, possible scores are 12–60; higher scores indicate greater growth. Coefficient alphas for the subscales were .83–.89 for grandparents.

### Grandparent Role Function (Employee, Spouse/Partner)

*Employee Functioning* measures grandparents’ ability to function in their jobs. Grandparents indicated whether they were employed before and/or after the grandchild’s death, whether they took time off from work post-death and if so, how much time they took off. On a scale of 1 “never” to 10 “all the time,” grandparents rated how often they had trouble focusing on work and how often they thought about their grandchild on the job in the previous 2 weeks.

The DAS [32] measures the *quality of the spouse/partner relationship* (cohesion, consensus, satisfaction, affectional expression) over the previous 2 weeks. Grandparents living with a spouse/partner rated the 32 items on Likert-type scales; possible total scores were 0–151. Higher scores indicate more favorable spouse/partner relationships. Total scale reliability was .93 for grandparents.

### Level of Childcare

Grandparents chose 1 of 6 phrases that best described the level of care they had provided to the grandchild before death. Grandparents’ choices were dichotomized as “no” (rarely/never, n = 95) and “yes” (babysitting to living with deceased grandchild, n = 40). Grandparents self-identified **race/ethnicity** as: Hispanic, White non-Hispanic, and Black non-Hispanic.

### Data Analysis

Scores on the measures of grief (Despair, Panic Behaviors, Blame and Anger, Disorganization, Detachment, Personal Growth), health (self-rated health, depression, PTSD), and role functioning (DAS total score, how often trouble focusing at work or thinking about the grandchild) were compared by race/ethnicity (Hispanic, Black non-Hispanic, White non-Hispanic) and level of childcare (rarely/never vs. babysitting to living with grandchild) with 2-way ANOVA and ANCOVA controlling for the number of weeks since the grandchild’s death. Differences in grandparent depression severity categories and presence/absence of PTSD by race/ethnicity and level of childcare were tested with Chi square analysis.

## Results

### Sample

Age range for the 136 grandparents was 36–77 (Table 1); of the 47 (35 %) over 60: 27 (57 %) were 60–65, 12 (26 %) were 66–70, and 8 (17 %) were 71–77. No grandparents were excluded for a low MMSE. Most were minority race/ethnicity (62 %), high school graduates (87 %), married or living with a partner (63 %), and employed (63 %). Half (52 %) had annual incomes less than $50,000.

**Table 1:** Description of grandparents

Characteristic	Grandparents (*N* = 136)	Grandmothers (*n* = 99)	Grandfathers (*n* = 37)
Age [*M* (*SD*)]	55.3 (9.8)	53.9 (10.2)	59.0 (7.6)
Race/ethnicity [*n* (%)]			
Black non-Hispanic	52 (38.2)	43 (43.4)	9 (24.3)
White non-Hispanic	52 (38.2)	31 (31.3)	21 (56.8)
Hispanic	32 (23.6)	25 (25.3)	7 (18.9)
Education [*n* (%)]			
<High school	18 (13.2)	15 (15.2)	3 (8.1)
High school grad	33 (24.3)	24 (24.2)	9 (24.3)
Some college or training	53 (39.0)	39 (39.4)	14 (37.9)
College graduate	32 (23.5)	21 (21.2)	11 (29.7)
Has a partner [*n* (%)]	85 (62.5)	54 (54.5)	31 (83.8)
Total income [*n* (%)]			
<$20,000	30 (22.1)	25 (25.3)	5 (13.5)
$20,000–49,999	41 (30.1)	34 (34.3)	7 (18.9)
≥$50,000	41 (30.1)	22 (22.2)	19 (51.4)
No response	24 (17.7)	18 (18.2)	6 (16.2)
Employed [*n* (%)]			
Before grandchild death	93 (68.4)	62 (62.6)	31 (83.8)
After grandchild death	86 (63.3)	57 (57.6)	29 (78.4)
Child care for deceased [*n* (%)]			
No	95 (69.9)	67 (67.7)	28 (75.7)
Yes	40 (29.4)	31 (31.3)	9 (24.3)
No response	1 (.7)	1 (1.0)	0 (0)

Mean age of the 115 deceased grandchildren was 12.8 months (*SD* = 20.71); 37 % were <1 month old (Table 2). Most were boys (65 %) and had died in the hospital (77 %). Prematurity and congenital/genetic conditions were the two most common causes of death. Two-thirds of grandparents expected the grandchild’s death. The deceased was the only grandchild for 22 (17 %) grandparents and the first born for 31 (25 %) grandparents. Most grandparents (70 %) had provided limited or no childcare for this grandchild; 12 grandparents had lived with this grandchild.

**Table 2:** Description of deceased grandchildren

Characteristic	Grandchildren (*N* = 115)	Boys (*n* = 75)	Girls (*n* = 40)
Age in months [*M* (*SD*)]	12.8 (20.71)	12.6 (20.96)	13.0 (20.49)
Age group [*n* (%)]			
<1 month	42 (37.0)	29 (39.0)	13 (32.5)
1–12 months	40 (35.0)	24 (32.0)	16 (40.0)
12.1–24 months	14 (12.0)	10 (13.0)	4 (10.0)
24.1–72 months	19 (16.0)	12 (16.0)	7 (17.5)
Race/ethnicity [*n* (%)]			
Black non-Hispanic	45 (39.0)	30 (40.0)	15 (37.5)
White non-Hispanic	29 (25.0)	19 (25.3)	10 (25.0)
Asian non-Hispanic^a^	2 (2.0)	2 (2.7)	0
Biracial non-Hispanic	4 (4.0)	2 (2.7)	2 (5.0)
Hispanic	35 (30.0)	22 (29.3)	13 (32.5)
Where died [*n* (%)]			
Hospital	88 (77.0)	60 (80.0)	28 (70.0)
Home	21 (18.0)	12 (16.0)	9 (22.5)
At scene, en route to hospital	6 (5.0)	3 (4.0)	3 (7.5)
Cause of death [*n* (%)]			
Prematurity	33 (28.7)	26 (34.6)	7 (17.5)
Congenital/genetic conditions	24 (20.9)	12 (16.0)	12 (30.0)
SIDS	15 (13.0)	10 (13.3)	5 (12.5)
Cancer	9 (7.8)	6 (8.0)	3 (7.5)
Injury	8 (7.0)	5 (6.7)	3 (7.5)
Infection	8 (7.0)	6 (8.0)	2 (5.0)
Kidney/multi-organ failure	4 (3.5)	2 (2.7)	2 (5.0)
Respiratory	3 (2.6)	3 (4.0)	0
Seizure	2 (1.7)	0	2 (5.0)
Iatrogenic	2 (1.7)	2 (2.7)	0
Don’t know	7 (6.1)	3 (4.0)	4 (10.0)

^a^Grandchildren were Asian, but grandparents are White non-Hispanic

### Grandparent Health

Grandparents’ self-ratings of health averaged 7.7 (*SD* = 2.06) on a 1–10 scale; 22 (16 %) grandparents rated their health as 5 or less. Main and interaction effects of grandparent race/ethnicity and level of childcare on self-rated health were not significant.

Most grandparents (68 %) reported having been diagnosed with a chronic health condition: 32 % with one, 36 % with two or more. Conditions diagnosed after the grandchild’s death included 10 mental health conditions, 4 with hypertension, 3 with angina, 1 with arthritis and 2 with cancer. A total of 19 medication changes were reported by 17 grandparents: 10 increased and 1 decreased the dosage; 3 added, 2 deleted and 3 switched medications. Since the grandchild’s death, 38 (28 %) grandparents reported a total of 59 illnesses, and 6 (4.5 %) reported a total of 7 hospitalizations. Thirty-one percent of grandparents reported symptomatology indicative of clinical depression (17 % mild, 14 % moderate/severe) and 35 % indicative of PTSD. Two-way ANOVAs and ANCOVAs indicated that grandparents who had provided some care for their deceased grandchild had significantly more severe symptoms of depression and PTSD (Table 3). Controlling for time since death, Black grandparents had significantly more severe PTSD symptoms than White grandparents. In addition, the interaction effect was significant for PTSD, controlling for time since death. For White and Black, but not Hispanic grandparents, PTSD symptoms were more severe if they provided care. Significantly more grandparents who provided care to their deceased grandchild had clinical depression, χ^2^ = 15.87, *df* = 2, *p* < .001, or PTSD, χ^2^ = 15.13, *df* = 1, *p* < .001, than grandparents who rarely/never provided care. Presence of clinical depression and PTSD were related, χ^2^ = 46.93, *df* = 2, *p* < .001; 27 (20 %) grandparents had both conditions. Only eight grandparents reported being in therapy: three with clinical depression and PTSD, one with PTSD only, and four with neither condition.

**Table 3:** Significant differences in grief, mental health, and role function by racial/ethnic and childcare group

	Racial/ethnic group	Level of childcare to deceased grandchild			Main effects			Interaction effect
		Total *M* (*SD*)	Rarely/never *M* (*SD*)	“Sometimes” to “Lived with” *M* (*SD*)	Model *F*	Childcare *F*	Race/ethnicity *F*	Child care × race/ethnicity *F*
Despair	Total		21.1 (7.57)	29.4 (13.00)	4.34**	14.44**	.29	.61
	Black	23.1 (9.18)	20.5 (7.46)	29.0 (10.21)				
	White	24.2 (11.56)	20.9 (6.92)	31.0 (15.90)				
	Hispanic	22.9 (9.43)	22.0 (8.74)	26.3 (12.14)				
Blame and Anger	Total		8.9 (3.18)	12.8 (4.28)	5.26**	7.28**	2.70	4.85**
	Black	10.1 (4.19)	8.9 (3.60)^a^	13.7 (6.34)^a^				
	White	10.1 (5.26)	8.7 (2.11)^b^	13.4 (8.17)^bc^				
	Hispanic	9.1 (3.53)	9.5 (3.88)	7.7 (1.25)^c^				
Detachment	Total		9.8 (3.05)	12.2 (6.13)	2.52*	5.19*	.82	1.70
	Black	10.4 (3.58)	9.7 (2.97)	11.9 (4.40)				
	White	10.9 (5.36)	9.7 (2.94)	13.5 (8.09)				
	Hispanic	10.3 (3.48)	10.3 (3.39)	10.1 (4.0)				
Depression	Total		8.0 (8.23)	14.7 (12.17)	3.47**	7.55**	1.23	1.99
	Black	10.9 (9.83)	8.6 (9.24)	16.0 (9.46)				
	White	9.9 (11.02)	7.0 (7.33)	16.4 (14.83)				
	Hispanic	8.6 (8.49)	8.7 (8.11)	8.1 (10.29)				
PTSD	Total		24.5 (18.25)	44.1 (23.28)	7.49**	7.05**	3.34*	3.77*
	Black	33.7 (22.44)	25.5 (18.96)^a^	52.3 (18.60)^ab^			Post hoc NS	
	White	24.4 (21.13)	19.0 (15.63)^bc^	39.4 (27.21)^c^				
	Hispanic	29.8 (18.13)	30.8 (19.07)	22.0 (1.73)				
Think about grandchild at work	Total		6.5 (2.82)	7.4 (2.82)	2.98*	2.57	4.38*	.22
	Black	7.4 (2.44)	7.0 (2.44)	8.2 (2.05)			Black > White	
	White	5.8 (2.59)	5.5 (2.63)	6.5 (2.42)				
	Hispanic	7.2 (3.14)	7.1 (3.28)	7.4 (2.82)				
Trouble focusing on work^d^ [M (SE)]	Total		3.6 (.29)	4.8 (.47)	2.33*	5.21*	.28	1.15
	Black	4.5 (.41)	3.5 (.46)	5.5 (.67)				
	White	4.1 (.41)	3.2 (.46)	5.1 (.69)				
	Hispanic	4.0 (.59)	4.0 (.56)	3.9 (1.04)				

* *p* < .05; ** *p* < .01

^a, b, c^Pairs of letters indicate significantly different groups

^d^Results for 2-way ANCOVA, controlling for time since death

### Grandparent Grief

Some grandparents reported having grown as persons since the grandchild’s death. Controlling for number of items on each HGRC subscale, Personal Growth had the highest mean of 3.53 (*SD* = .88), followed by Despair (*M* = 1.81, *SD* = .78), Disorganization (*M* = 1.75, *SD* = .83), Panic Behaviors (*M* = 1.58, *SD* = .60), Blame and Anger (*M* = 1.42, *SD* = .64), and Detachment (*M* = 1.32, *SD* = .54). Cutoff scores for HGRC subscales to categorize the severity of grief have not been established. To put the grandparents’ scores into context, they were compared to parents’ HGRC scores obtained within 6 months of their infant’s/child’s death in the Neonatal or Pediatric Intensive Care Unit [33]. Grandparents’ scores were significantly lower than parents’ scores on Despair, *t*(314) = 3.68, *p* < .001; Panic, *t*(338) = 3.70, *p* < .001; Blame and Anger, *t*(339) = 3.57, *p* < .001; and Detachment, *t*(342) = 5.22, *p* < .001. Grandparents scored significantly higher than parents on Personal Growth, *t*(340) = 1.96, *p* = .05. Their Disorganization scores were similar, *t*(351) = 1.78, *p* = .08.

Two-way ANOVAs were statistically significant for three HGRC subscales: Despair, Blame and Anger, and Detachment (Table 3). Grandparents who had provided some childcare for the deceased had significantly more Despair, Blame and Anger, and Detachment than those who rarely/never provided care for this grandchild. Interaction effects of race/ethnicity and childcare were significant for one subscale. Black grandparents and White grandparents who had provided some childcare for the deceased had significantly more Blame and Anger than Black grandparents and White grandparents who never/rarely provided care to this grandchild, respectively. White grandparents who provided some childcare had more Blame and Anger than Hispanic grandparents. The ANOVAs for Panic, *F*(5,125) = .73, *p* = .60; Disorganization, *F*(5,127) = 1.36, *p* = .25; and Personal Growth, *F*(5,119) = 2.15, *p* = .06 were not significant. Controlling for number of weeks post-death at grandparent interview did not change these findings.

### Role Function

Of the 93 (68 %) grandparents who were employed before the grandchild’s death, 85 (91 %) returned to their jobs after the death and 8 (9 %) did not. Of the 43 (32 %) grandparents not employed before the death, 42 remained not employed and one grandparent became employed post-death. Most grandparents (80 % of grandmothers, 45 % of grandfathers) took time off from work. All but 12 of the 86 employed grandparents returned to work by 14 days post-death.

Two-way ANOVAs for grandparents’ ratings of how often they had trouble focusing on their work (controlling for time since death) and how often they thought about their grandchild on the job were statistically significant (Table 3). Controlling for time post-death, grandparents who had provided some childcare for this grandchild had significantly more trouble focusing on their work than grandparents who rarely/never provided childcare. When on the job, Black grandparents thought about their deceased grandchild significantly more often than White grandparents, regardless of time since the death. Interaction effects for level of childcare by race/ethnicity were not significant.

Comparisons of grandparents’ DAS scores by race/ethnicity and level of childcare were not statistically significant with and without control for time post-death.

## Discussion

In the first year after their young grandchild’s death, 63 (46 %) grandparents had clinical depression or PTSD or both. The 31 % depression rate in this study’s bereaved grandparents is considerably higher than the US depression rate of 7 % for adults 40–59 years old and 4 % for those 60 and older [34]. Only 6 % of this study’s 63 bereaved grandparents with depression, PTSD, or both, were in counseling at the time of the interview. This rate is much lower than the national rate of 24 % for those with moderate and 39 % for those with severe depression [34]. Perhaps this reflects grandparents’ concerns about the legitimacy of their grief for a deceased grandchild [10]. The study’s cross-sectional design prevented examination of the grandparents’ pattern of depression and PTSD. Longitudinal research is needed to characterize bereaved grandparents whose depression and/or PTSD does not resolve within the first year post-death.

Grandparents often are at an age when they may be more vulnerable to illness; 38 of the study’s grandparents reported a total of 59 illnesses in the 6 months following their grandchild’s death. Two-thirds reported at least one chronic health condition. Unlike bereaved parents [16], most of these chronic health conditions were diagnosed prior to the grandchild’s death. This is not surprising given that development of chronic conditions is more likely as one ages. Grandparents’ chronic conditions diagnosed post-death included mental health problems, hypertension, angina, and cancer, conditions found to be stress-related and consistent with studies of bereaved parents who also have increases in these types of chronic conditions [1–3, 16, 35, 36]. Grandparent-reported changes in medication regimens suggest that grandparents’ chronic conditions were more difficult to control after their grandchild’s death. Anecdotally, a number of grandparents reported not taking their medications for hypertension, angina, asthma, and other conditions for financial reasons.

Grandparents experienced a level of disorganization—difficulty concentrating, remembering, learning new things—in the first 6 months after their young grandchild’s death that was similar to that of parents within 6 months of their infant’s/child’s NICU/PICU death. The shock, numbness, and disbelief of a grandchild’s death likely contribute to this commonly reported feeling of disorganization. During the first 6 months post-death, many activities occur on behalf of the deceased—funeral, cremation, and burial arrangements; financial obligations (paying medical, hospital, and related bills); filing for health care and life insurance benefits; cancelling or changing scheduled activities and appointments; among others. Grandparents may need to change or cancel anticipated activities and/or plans for outings, an upcoming holiday, or a family event. The death also may disrupt established routines between the grandparent and grandchild. These changes may leave grandparents, like parents, feeling disorganized.

Most grandparents employed before the death returned to work within 2 weeks post-death, perhaps to stay busy [37] or because many employees lack paid time off for a grandchild’s death [26]. Most bereaved fathers also returned within 2 weeks and most bereaved mothers within 4 weeks [16]. Grandparents who had provided care to the deceased grandchild had more trouble concentrating on work. The short period of time that grandparents had to grieve for their grandchild and their adult child (grandchild’s parent) before returning to their jobs may exacerbate this problem.

In the US, many grandparents live with and/or are responsible for their grandchildren. Although more White grandparents live with their grandchildren, more Black live-in grandparents are responsible for their grandchildren [22]. Many grandparents who do not live with their grandchildren provide a considerable amount of childcare for them. In this study, 33 % Black, 31 % White, and 23 % Hispanic grandparents provided some level of childcare for their deceased grandchild—from occasional to full custody—and 68 % rarely/never provided childcare to this grandchild. Perhaps the grandchild’s very young age at death (37 % died in the first month of life) decreased grandparents’ opportunities to take care of this grandchild. It may also reflect grandparents continuing their employment into their 70s [38], the geographic mobility and separation of US families, and the resulting isolation of grandparents from their children and grandchildren. Approximately 68 % of the study’s grandparents were employed before their grandchild’s death, and many lived in a different state or country from their deceased grandchild.

Depression and PTSD were related to both childcare and race/ethnicity. Caregiving grandparents experienced significantly more severe symptoms of depression and PTSD than non-caregiving grandparents. Caregiving grandparents may feel a greater sense of responsibility and have more changes in their daily lives due to the grandchild’s death, affecting their mental health more than non-caregiving grandparents. However, caregiving Hispanic grandparents had less severe PTSD symptoms than caregiving Black and White grandparents. Hispanic grandparents who did not provide childcare may feel more distress than Hispanic grandparents who did, thinking they had not met their cultural obligations to the family [39].

Childcare and race/ethnicity also played a part in the level of grandparents’ grief. Grandparents who had provided some childcare for the deceased experienced more despair, blame and anger, and detachment than those who rarely/never provided care for this grandchild, especially for Black and White grandparents. As with mental health, the greater time that grandparents spend with their grandchild for caregiving may result in stronger feelings of responsibility for the grandchild and more daily experiences, memories, or situations to trigger their grief. Hispanic caregiving grandparents reported less blame and anger, but not despair or detachment, than White caregiving grandparents. Perhaps this is because Hispanic families value a focus on the family as a whole rather than on the individual [40]. Grandparents who had provided care for the grandchild may be more closely tied into the family and able to share their grief.

Grandparents who provided care for their deceased grandchild reported more trouble focusing on work than grandparents who did not provide childcare, but only when the influence of time since death was controlled. Time since death may be important if it has an influence on the intensity of PTSD symptoms, especially intrusive thoughts of the grandchild, since caregiving grandparents also had more severe PTSD symptoms. Black grandparents reported thinking about their deceased grandchild at work more often than White grandparents, regardless of time post-death. This is consistent with the finding of more intense PTSD symptoms for Black grandparents than White grandparents.

Grandparents clearly are adversely affected by their grandchild’s death, especially when they had provided care for that grandchild before death. Unlike bereaved parents, bereaved grandparents rarely receive attention and help from health care providers, family, and friends [9, 10, 13]. Grandparents are in a precarious position with both their own health and their relationship with their adult child [11]. Health care providers need to treat grandparents as they do parents. All visits to a health care provider should include questions about deaths that grandparents may have experienced since their last visit. Those reporting a grandchild’s death, wherever possible, should be linked to healthcare, social, and religious organizations offering grief counseling focused on the unique experiences of grandparents bereft of a grandchild. Incorporating them into intergenerational bereavement groups has been reported to be important in their coping with their loss [14]. Assessment of the impact of a grandchild’s death on grandparent health, grief and functioning, with appropriate referrals for physical and mental health interventions, is important. The present study’s data indicate few grandparents are in counseling following their grandchild’s death although about one-third had clinically significant symptoms of depression and/or PTSD.

Longitudinal research is needed to determine grandparent health, grief and role functioning changes over time following grandchild death. Stress-related health outcomes, including cancer, heart disease, mental disorders and deaths, increase for bereaved parents 1–5 years after loss of a child [1–4]. No such longitudinal data have been reported for grandparents who are at a more vulnerable time in life. Such data would provide guidance for developing efficient and cost effective interventions targeted to those grandparents most at risk of poor health outcomes.
